# Retraction: Comparative Expression Profile of miRNA and mRNA in Primary Peripheral Blood Mononuclear Cells Infected with Human Immunodeficiency Virus (HIV-1)

**DOI:** 10.1371/annotation/d28d38b2-41a3-42a6-b421-68f9460a676d

**Published:** 2012-08-29

**Authors:** Ankit Gupta, Pruthvi Nagilla, Hai-Son Le, Coulton Bunney, Courtney Zych, Anbupalam Thalamuthu, Ziv Bar-Joseph, Sinnakaruppan Mathavan, Velpandi Ayyavoo

The authors wish to retract this article for the following reason:

Upon re-evaluation of the analyses performed, we discovered an error in the data fed into the software, which resulted in incorrect results in Table 2 and Figure 2. During the initial analysis, we eliminated miRNAs if they showed an expression CT of value 35 in over 75% of the samples. This decision was based on the instructions from the software during the initial data feed process for the selection of particular miRNAs (row) for exclusion. Unfortunately, the software included the excluded miRNAs as controls along with the endogenous controls and analyzed the data. As a result, the analyses identified miRNAs that are not statistically significant.

